# The plant cell cycle: Pre-Replication complex formation and controls

**DOI:** 10.1590/1678-4685-GMB-2016-0118

**Published:** 2017-03-16

**Authors:** Juliana Nogueira Brasil, Carinne N. Monteiro Costa, Luiz Mors Cabral, Paulo C. G. Ferreira, Adriana S. Hemerly

**Affiliations:** 1 Instituto de Bioquímica Médica Leopoldo de Meis, Universidade Federal do Rio de Janeiro, Rio de Janeiro, RJ, Brazil.; 2 Departamento de Biologia Celular e Molecular, Universidade Federal Fluminense, Niteroi, RJ, Brazil.; 3 Centro Universitário Christus, Fortaleza, CE, Brazil.; 4 Centro de Genômica e Biologia de Sistemas, Universidade Federal do Pará, Belém, PA, Brazil.

**Keywords:** Pre-replication complex, A. thaliana, cell cycle

## Abstract

The multiplication of cells in all living organisms requires a tight regulation of DNA replication. Several mechanisms take place to ensure that the DNA is replicated faithfully and just once per cell cycle in order to originate through mitoses two new daughter cells that contain exactly the same information from the previous one. A key control mechanism that occurs before cells enter S phase is the formation of a pre-replication complex (pre-RC) that is assembled at replication origins by the sequential association of the origin recognition complex, followed by Cdt1, Cdc6 and finally MCMs, licensing DNA to start replication. The identification of pre-RC members in all animal and plant species shows that this complex is conserved in eukaryotes and, more importantly, the differences between kingdoms might reflect their divergence in strategies on cell cycle regulation, as it must be integrated and adapted to the niche, ecosystem, and the organism peculiarities. Here, we provide an overview of the knowledge generated so far on the formation and the developmental controls of the pre-RC mechanism in plants, analyzing some particular aspects in comparison to other eukaryotes.

## Introduction

The eukaryotic cell cycle is a highly coordinated process, when a cell replicates its genome and divides it equally into two daughter cells. In order to assure that the genome will not only be correctly duplicated, but also correctly divided between daughter cells, a great number of control mechanisms take place during the cell cycle events. DNA replication is tightly monitored to ensure that the genome is replicated just once per cell cycle (Kearsey and Cotterill, 2003). This control relies on a mechanism that takes place before cells enter S phase and licenses cells for replication by selecting and activating origins of replication. The system is formed by the sequential recruitment of proteins to DNA replication origins, establishing the pre-replication complex (pre-RC). It represents the key process in controlling chromosome replication.

The “permission to replicate” is directly connected to a number of internal and external features, like the availability of nutrients, cell size, and others, in a way that cells can decide between entering S phase (thus initiating the process of cell division) or exit cell division cycle and start differentiating. The external controls regulating this step of the cell cycle are diverse among the multicellular eukaryotes, following their different developmental strategies, and interfering in the mechanisms regulating pre-RC activity. Therefore, this review uncovers the knowledge generated so far on the formation and the developmental controls of the pre-RC machinery in plants, analyzing some particular aspects in comparison to other eukaryotes.

## Plants as a model for studies on DNA replication controls.

**Figure 1:**
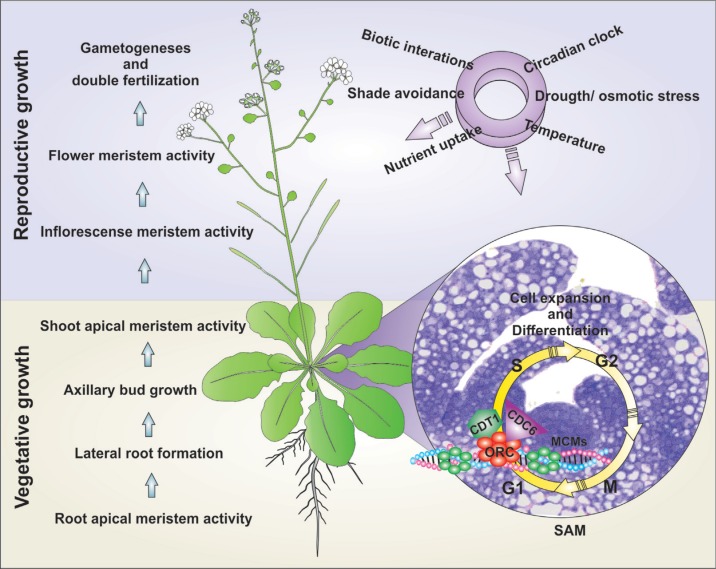
Overview of cell cycle control modulation at meristems by endogenous and exogenous signals. Plants are continuously sensing the environment and modulating their development by adjusting cell division and differentiation rates at the different meristems. This means that every plant meristem might be sensing exogenous signals and integrating with genetic controls, which leads to changes in gene expression that will finally balance cell proliferation and differentiation rates, culminating with the correct plant form. The shoot apical meristem (SAM) is represented in the right panel. An important control of the G1 to S transition of the cycle is the pre-replication complex (pre-RC) that might be continuously regulated, although by some different mechanisms, along development.

Plants are good models to study DNA replication controls, since they have various developmental and genome particularities. Plant development, compared to animals, is highly influenced by the environment in which they grow, suggesting that plants have evolved specific mechanisms that convey environmental signals to control cell division and ultimately plant growth. Plants, via post-embryonic organogenesis (Inzé and De Veylder, 2006), form new organs during their complete life span, and this continuous formation is tightly connected with the surrounding environment (Figure 1). Early in the development of the embryo, polarized forces establish the root and shoot meristems (Scheres, 2007). These meristematic cells and their descendants give rise to the various tissues and organs of a mature plant through the combined processes of cell division, cell expansion and cell differentiation. This ability depends on both the maintenance of proliferating cells in the meristems and the re-initiation of cell division in non-dividing cells (Veylder *et al.*, 2003). Considering that plant cells don’t have the ability to move through the plant body, it becomes extremely important to coordinately control cell division and differentiation, so that a given cell can be present at its final place with its definitive fate (Brukhin and Morozova, 2011). In addition, higher plants adopt particular strategies of development ending up with a great variability of body architectures. This suggests that signaling controls regulating individual steps of the basic DNA replication machinery might also differ among plant species.

The plant kingdom is divided in two large groups: the monocots and the dicots. The best-studied members of each group are the dicot *Arabidopsis thaliana* and the monocot *Oryza sativa*. Although they share many characteristics, some important differences in their developmental plans and genome structure are present.

Different from other kingdoms, higher plants present extreme differences in its genome size (followed by phenotypical differences), with variations of more than 2,500 fold (Grime and Mowfforth, 1982). Allopolyploids, which are organisms that inherit their chromosomes from different species, are also common. Although chromosome number within species is usually constant, it can vary among plant species in a range that goes from n = 2 (in *Haplopappus gracilis*) to n = 100 (in *Senecio biserratus*). And, more interestingly, this number can also vary widely from generation to generation. Remarkably, it is also well known that controls coupling DNA replication with mitosis are quite flexible in plants. During development, plant cells often modify their classical cell cycle and undergo endoreduplication events that allow them to increase their ploidy level (Sugimoto-Shirasu and Roberts, 2003). However, the consequences of this modified cell cycle for plant development are not completely understood.

Taken together, all these characteristics indicate that plants developed a number of novel regulatory networks to integrate cell cycle progression, cell growth and differentiation with endogenous and exogenous signaling, becoming unique organisms for the study of DNA replication and developmental abilities (Boniotti and Griffith, 2002). Thus, there has been an increasing interest in investigating the relationship between DNA replication controls and development in higher plants, and in the comparison with mechanisms employed by other eukaryotes. In this context, the first step that licenses DNA is one important crossroad when internal and environmental signals are integrated with cell division in order to trigger the developmental program in a flexible way.

## Licensing DNA replication: the assembly of the Plant pre-Replication Complex

### Plant DNA Origins

Cells must license DNA for replication by selecting and activating specific origins of replication. In eukaryotes, there is a large number of possible origins of replication, but only a group of these available origins is chosen to be used by different types of cells and in different moments during development. Origin activation is possibly the result of a multisource signaling pathways where positive and negative proliferating signals result in a response of the pre-RC machinery, in a way that once the origin activation starts cells are compromised with DNA replication.

The origins of replication consist of DNA sequences or chromatin marks (or both) that are recognized by proteins that bind to DNA, the Origin Recognition Complex (ORCs), and where other proteins will also bind to form the pre-RC (Cunningham and Berger, 2005).

In budding yeast, the recognition of origins by ORC seems to be unique because a consensus sequence has already been determined (the A-rich sequences named ACS) (Bell and Dutta, 2002; Nieduszynski *et al.*, 2006). In contrast, it has been difficult to find clear consensus sequence in multicellular eukaryote organisms, due to their large genome size and fluctuation during differentiation and development (Aladjem, 2007; Hiratani *et al.*, 2009). From *Drosophila* studies came the intriguing observation that the number of useful replication sites in a genome depends on other factors besides nucleotide sequence. In rapidly dividing nuclei of the *Drosophila* zygote there are many more initiation sites than in cultured cells, in a way that replication origins used during the early stages of embryogenesis are not used by mature cells (Blumenthal *et al.*, 1974). Another possibility is that pre-RC interaction with the DNA might be related with DNA structure rather than sequence, as indicated in recent structural studies on archaeal orthologs of ORC interacting with the origin recognition box (Gaudier *et al.*, 2007).

Experimental difficulties are the main constraint in the identification of a plant replication origin and in the characterization of its mechanism of recognition during DNA replication (Lee *et al.*, 2009; Shultz *et al.*, 2009; Costas *et al.*, 2011). Although little is known about DNA replication origins in plants, some features can be pointed as specific for plant origins in comparison to other eukaryotes (Bryant and Aves 2011; reviewed in Raynaud *et al.*, 2014). It seems that origin consensus sequences are more GC-rich in metazoans and plants (Costas *et al.*, 2011; Bass *et al.*, 2014), in contrast with the AT-rich sequences in yeast (Mechali *et al.*, 2013). Localization of these origins is preferentially near promoters of genes in metazoans (Cayrou *et al.*, 2011). In *A. thaliana*, a study has sequenced and identified ~1,500 putative genome-wide origins (Costas *et al.*, 2011). That work revealed that 77.7% of origins were co-localized with gene units, preferentially towards their 5’ end. Also, highly expressed genes tended to have more origins in regions immediately upstream or downstream (Costas *et al.*, 2011). Replication origins from metazoans and yeast were found to be enriched with epigenetic marks (Dorn and Cook, 2011). In the same way, *A. thaliana* replication origins were found to be enriched with H3K4me3, H4K5ac and the variant histone H2A.Z (Costas *et al.*, 2011). Also, it is possible that chromatin modification proteins are needed to specify and/or activate origins in *A. thaliana*, as (1) it has been shown that two HATs (histone acetyltransferases) are redundantly required for gametophyte development (Latrasse *et al.*, 2008); and (2) origins of chromosome 4 are associated with H3Lys36ac (Lamesch *et al.*, 2012). The same logic was found in animals: HATs are required to stabilize chromatin for ORC loading in *Drosophila* (Vorobyeva *et al.*, 2013), and some chromatin modifier proteins are needed for the correct assembly or activation of pre-RC in mammals (Tardat *et al.*, 2010) and yeast (Rizzardi *et al.*, 2012).

### How is the pre-RC assembled in plants?

**Figure 2:**
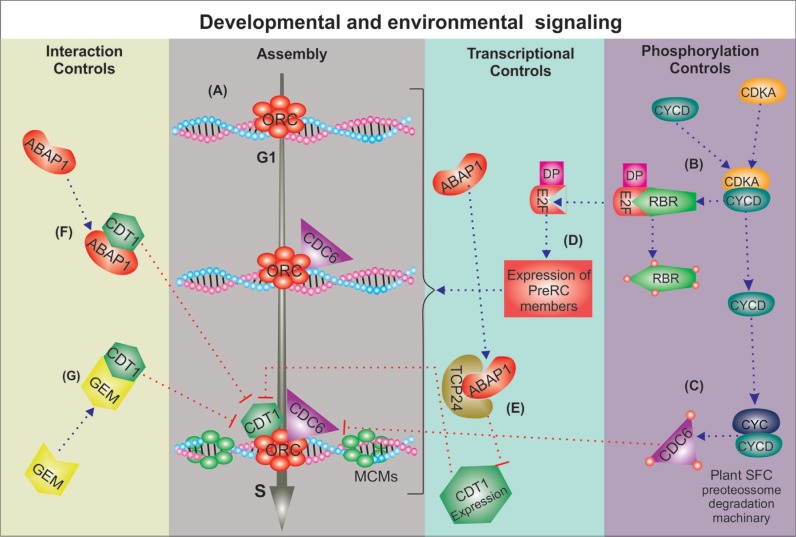
Hypothetical model of pre-RC formation and regulation in plants. (A) Pre-replication complex (Pre-RC) assembly, activation and prevention of DNA re-replication is regulated by three major levels of controls that act in a coordinated way, connecting cell cycle progression with endogenous and exogenous (environmental) signaling. Protein phosphorylation by CDK/cyclin regulates different steps of DNA replication licensing: (B) first, they phosphorylate retinoblastoma protein, releasing E2F/DP to (D) activate transcription of pre-RC genes; (C) later, phosphorylation of members of the pre-RC promotes initiation of DNA replication and prevents DNA re-replication through nuclear exclusion and/or protein degradation. In addition to the transcriptional regulation by E2F/DP (D), pre-RC assembly is limited by repression of CDT1 transcription (E) by ABAP1/TCP24 (F), which also interacts directly with CDT1. (G) GEM also competes for binding to CDT1 making it less available for pre-RC loading.

A generalized eukaryotic licensing model based on yeast and animal systems support that origin selection begins at the transition from M to G1 phases of the cycle. At this moment, mitogenic signaling from the environment triggers the expression of Cyclin D, a protein that interacts with CDKA (cyclin-dependent kinase A), forming the CDKA/CyclinD complex (Dresselhaus *et al.*, 2006). This complex promotes phosphorylation of different targets, including the retinoblastoma protein (RB), releasing the transcription factor E2F to promote expression of many pre-RC genes that bind to DNA replication origins at G1 phase (Figure 2). The first event in the formation of the pre-RC is the assembly of the ORC – a complex of six conserved subunits (ORC1–ORC6) to replication origins (Bell, 2002). After ORC assembly, other members of the pre-RC use ORC as a landing platform, to which they bind. The recruitment of CDC6 by ORC is the next step in pre-RC assembly, followed by the recruitment of CDT1 (Bell and Dutta, 2002). In addition, CDC6 and CDT1 proteins act synergistically to load a complex formed by six proteins – the MCM complex (MCM2–7; Mini Chromosome Maintenance). MCM loading is the last event in the licensing mechanism. After that, origins are licensed to replicate and any site containing the MCM complex has the potential to form an active DNA replication fork (Masai *et al.*, 2005) (Figure 2). The resulting complex loaded onto DNA, consisting of ORC1-6/CDC6/MCM2-7, is termed the pre-RC (for evolutionary aspects of pre-RC proteins through Archaea to eukaryotes see Bryant and Aves, 2011).

Pre-RC members can be identified by sequence homology in all genomes of higher plants available in public databases. A great number of members, were reported in eudicot *A. thaliana* (Gavin *et al.*, 1995; Collinge *et al.*, 2004; Masuda *et al.*, 2004), and monocots like *O. sativa* (Kimura *et al.*, 2000; Li *et al.*, 2005; Mori *et al.*, 2005; Shultz *et al.*, 2007), and *Zea mays* (Sabelli *et al.*, 1996, 1999; Bastida and Puigdomenech, 2002; Witmer, 2003; Dresselhaus *et al.*, 2006) (Table 1). These features suggest that pre-RC function has been conserved in the course of eukaryote evolution, and support the belief of a high conservation of the replicative machinery among plants.

**Table 1:** Pre-replication complex (pre-RC) genes published in literature in main plants models. The numbers in the table correspond to the numbers of homologs found in the organisms. Note that here we accounted only for genes cloned and characterized, although others could be found by sequence similarities by BLAST search in genomic data bases available online. At: *A. thaliana;* Os: *Oryza sativa;* Zm: *Zea mays*.

Protein	ID	*A. thaliana*	ID	*O. Sativa*	ID	*Z. mays*
ORC1	At4g14700/	BAH domain, PHD zinc finger domain, AAA_ATPase type	LOC_Os04g10650	BAH domain, AAA_ATPase Type	GRMZM2G035665/GRMZM2G032209/GRMZM2G004924	BAH domain, AAA_ATPase Type, P-loop_NTPase domain
	At4g12620					
ORC2	At2g37560	-	LOC_Os10g34820	-	GRMZM2G117238	-
ORC3	At5g16690	-	LOC_Os10g26280	-	GRMZM2G381822	-
ORC4	At2g01120	AAA_ATPase type	LOC_Os01g49010	AAA_ATPase type	GRMZM5G876520	-
ORC5	At4g29910	-	LOC_Os03g55200	-	GRMZM2G089556	
ORC6	At1g26840	-	LOC_Os07g43540	-	GRMZM5G825512	
CDC6	At2g29680/	-	LOC_Os01g63710	P-loop NTPase domain, AAA_ATPase type	GRMZM2G007659/GRMZM2G363408	-
	At1g07270					
CDT1	At2g31270/	-	LOC_Os04g10650/LOC_Os10g34820	-	GRMZM2G035665/GRMZM2G032209/GRMZM2G004924	-
	At3g54710					
MCM2	At1g44900	MCM N-Term superfamily, P-loop_NTPase domain, OB-fold domain	LOC_Os11g29380	MCM N-Term superfamily, P-loop_NTPase domain, AAA_ATPase type	GRMZM2G139894	MCM N-Term superfamily, P-loop_NTPase domain, AAA_ATPase type
MCM3	At5g46280	MCM N-Term superfamily, P-loop_NTPase domain, OB-fold domain, AAA_ATPase type	LOC_Os05g08100	MCM N-Term superfamily	GRMZM2G162445/GRMZM2G100639	MCM N-Term superfamily, P-loop_NTPase domain, AAA_ATPase type
MCM4	At2g16440	MCM N-Term superfamily, P-loop_NTPase domain, OB-fold domain, AAA_ATPase type	LOC_Os01g36390	MCM N-Term superfamily, P-loop_NTPase domain, AAA_ATPase type	GRMZM2G066101	MCM N-Term superfamily, P-loop_NTPase domain, AAA_ATPase type
MCM5	At2g07690	MCM N-Term superfamily, P-loop_NTPase domain, OB-fold domain	LOC_Os02g55410	MCM N-Term superfamily	GRMZM2G075978	MCM N-Term superfamily, P-loop_NTPase domain
MCM6	At5g44635	MCM N-Term superfamily, P-loop_NTPase domain, OB-fold domain	LOC_Os05g14590	MCM N-Term superfamily, P-loop_NTPase domain	GRMZM2G021069	MCM N-Term superfamily, P-loop_NTPase domain
MCM7	At4g02060	MCM N-Term superfamily, P-loop_NTPase domain, OB-fold domain, AAA_ATPase type	LOC_Os12g37400	MCM N-Term superfamily, P-loop_NTPase domain, AAA_ATPase type	GRMZM2G065205	MCM N-Term superfamily, P-loop_NTPase domain, AAA_ATPase type

Nonetheless, some differences can be found in pre-RC members among plant species. Different from other eukaryotes studied so far, *A. thaliana* houses in its genome two homologs of *ORC1*, *CDT1* and *CDC6* (Ramos *et al.*, 2001; Collinge *et al.*, 2004; Masuda *et al.*, 2004; Diaz-Trivino *et al.*, 2005; Raynaud *et al.*, 2005). Two *OsCDT1* homologs are also present in the monocot rice (Shultz *et al.*, 2007), but these gene duplications are not necessarily found in all plant species, suggesting that it is not a general feature in the plant kingdom (Table 1).

*AtORC1* homologs, called *ORC1a* and *ORC1b,* are highly similar proteins, with around 90% of amino acid similarity. The N-terminal portion of *AtORC1a* and *AtORC1b* contains a BAH (Bromo-Adjacent Homology) domain and a PHD (plant Homeodomain). BAH, associated with a PHD, has been implicated in linking DNA methylation, replication, and transcriptional regulation in mammals (Aasland *et al.*, 1995; Callebaut *et al.*, 1999). ORC1 has already been described as a transcriptional regulator in plants (Pak *et al.*, 1997; Saitoh *et al.*, 2002). The presence of the PHD exclusively in plant *ORC1* genes among all other eukaryotes creates a very interesting observation, once this domain is responsible for specific binding to H3K4me3, both *in vitro* and *in* vivo (Sanchez and Gutierrez, 2009). Thus, it is possible that plant *ORC1* genes are playing an important role on epigenetic regulation of cell cycle and plant development.

One copy of each *MCM* gene has been identified in *A. thaliana* (Springer *et al.*, 1995; Stevens *et al.*, 2002; Masuda *et al.*, 2004; Schreiber *et al.*, 2006). Also, *OsMCM2* encodes a functional homologue of the CDC19 fission yeast protein, able to rescue the wild-type phenotype in a mutant yeast for this gene, demonstrating how structurally close these proteins are (Cho *et al.*, 2008). Finally, it has been shown that all six *MCM* cDNAs from pea (*Pisum sativum*) contain Zinc Finger motifs, ATPase consensus sequences like Walker A motif, Walker B motif or MCM signature, and an Arginine Finger motif (Tran *et al.*, 2010). *PsMCM2* contains a putative nuclear localization sequence, which is required for entering into the nucleus during G1/S-phase*. PsMCM4* has an endoplasmic reticulum target and phosphorylation sites for CDKs. *PsMCM5* contains the leucine zipper pattern consensus sequence, present in regulatory proteins including transcription factors (Tuteja *et al.*, 2011) (Table 1).

It is interesting to observe that the core DNA replication machinery components in plants are, by sequence homology, more similar to human than to budding yeast (Shultz *et al.*, 2007). Moreover, some *A. thaliana* pre-RC members did not show any significant alignment with budding yeasts. This also confirms the observation that some cell cycle proteins that are present in humans, but not in yeast, are also present in *A. thaliana* and rice (Shultz *et al.*, 2007).

## Regulation of pre-RC licensing by protein interactions, stability and distribution.

Although mechanisms of cell division are conserved in eukaryotes, plants contain a larger number of cell cycle regulatory genes, such as 71 cyclin genes identified in *A. thaliana*, while only 15 were described in yeast and 23 in human (Van Leene *et al.*, 2010). The understanding of how this complex machinery works involves revealing protein expression, interaction partners, targets and biological functions.

### Protein interactions between pre-RC members and beyond

In order to investigate the arrangement of the pre-RC components within the complex, studies on the characterization of physical interactions among the *A. thaliana*, rice and maize ORC subunits were performed by yeast two-hybrid and pull-down assays. Similarly, in these three plant species, ORC3 plays a central role in maintaining the complex associations; however, there are some intrinsic differences in ORC interaction patterns. In *A. thaliana,* protein interaction assays suggest a pre-RC architecture, in which AtORC3 and AtORC2 are central elements in maintaining the complex associations (Masuda *et al.*, 2004; Diaz-Trivino *et al.*, 2005). *A. thaliana* has two ORC1 subunits, and AtORC3 interacts only with AtORC1b and not with AtORC1a (Masuda *et al.*, 2004; Diaz-Trivino *et al.*, 2005). In rice, OsORC2 and OsORC3 are also central pre-RC subunits, forming a core that binds OsORC5 and recruits OsORC4 (Tan *et al.*, 2013). In maize, ZmORC3 can bind to ZmORC2, -4, -5, but ZmORC5 cannot bind to ZmORC2. Also, ZmORC1 has a weak interaction with the others (Witmer, 2003). In general, the interactions among plant pre-RC members are very similar to those found in humans (Dhar *et al.*, 2001). Intrinsic differences in ORC interaction patterns among closely related species may suggest that: (1) they have little impact on assembly and function, or (2) they might hide evolutionary strategies in DNA replication initiation among species (Tan *et al.*, 2013). Differences in expression profile among the pre-RC genes in the same plant species suggest that they might form different sub-complexes in the various plant tissues (discussed below). One interesting question that remains to be elucidated is whether the architecture of plant pre-RCs varies in different plant tissues and in response to developmental and environmental signals, acquiring specific roles depending on the developmental context.

Interactions of plant pre-RC members with proteins outside the complex have been identified by co-immunoprecipitation, pull-down and two-hybrid assays (Table 2). Nevertheless, few regulators of the plant DNA licensing machinery in response to cell differentiation and environmental signaling have been studied so far. Other than the cell cycle regulation by CDK-Cyclins, novel regulatory proteins have been described to control pre-RC assembly and/or function in plant development.

**Table 2:** Pre-replication complex (pre-RC) protein interactions observed in experimental analysis in *A. thaliana*. Techniques: 2HD: yeast two-hybrid PD: pull-down; IP: immuno-precipitation; TAP: tandem affinity purification.

Gene 1	Genes 2	Techniques	Reference
ORC1a (AT4G14700)	ABAP1 (AT5G13060)	2HD, PD, IP	Masuda *et al.*, 2008
	ABAP1 and TCP24 (AT1G30210)*	PD	Masuda *et al.*, 2008
	ORC3	PD	Masuda *et al.*, 2008
	DEA(D/H)-box (AT4G16630), EIF2 (AT5G20920), TIF3B1 (AT5G27640)	TAP	Van Leene *et al.*, 2010
ORC1b (AT4G12620)	ABAP1	2HD, PD	Masuda *et al.*, 2008
	ORC2 (AT2G37560), ORC5 (AT4G29910), ORC6 (AT5G42480)	PD	Diaz-Trivino *et al.*, 2005
ORC3 (AT5G16690)	ORC2, ORC4, ORC5, ORC6	PD	Diaz-Trivino *et al.*, 2005
	ORC1A	PD	Masuda *et al.*, 2008
	ABAP1	2HD, PD, IP	Masuda *et al.*, 2008
	CYCb1;4 (AT2G26760)	TAP	Van Leene *et al.*, 2010
CDT1a (AT2G31270)	ABAP1	2HD, PD, IP	Masuda *et al.*, 2008
	GEM (AT2G22475)	2HD, PD	Caro *et al.*, 2007
	CDKA, CYCD	PD	Castellano *et al.*, 2004
CDT1b (AT3G54710)	ABAP1	2HD, PD	Masuda *et al.*, 2008
	ABAP1 and TCP24*	PD	Masuda *et al.*, 2008
	ARC6	BiFC	Raynaud *et al.*, 2005
CDC6 (AT2G29680)	CDC2 (AT3G48750)	2HD	Pusch *et al.*, 2012
	UBQ3 (AT5G03240)	TAP	Kim *et al.*, 2013
	DUF936 (AT2G31920), AT4G28230, Histone H4, Cand1 (AT2G02560)	TAP	Van Leene *et al.*, 2010
MCM2, MCM 3, MCM4, MCM5	ETG1 (AT2G40550)	TAP	Van Leene *et al.*, 2010
MCM6 (AT5G44635)	ETG1 (AT2G40550), MCM2 (AT1G44900), ETG1, MCM4, MCM5, MCM7, RPN7 (AT4g24820), NMD3 (AT2G03820), eIF-2B (AT2G05830), EIF2 (AT5G20920), AT4G24820, SAMBA (AT1G32310), PPR-like (AT1G05670), IMPA1 (AT3G06720) AT2G05830, IMPa2 (AT4G16143), NAD7 (ATMG00510), AT-IMP (AT3G06720)	TAP	Van Leene *et al.*, 2010
MCM7 (AT4G02060)	GRF2 (AT1G78300)	TAP	Chang *et al.*, 2009
	ETG1 (AT2G40550), CEL3 (AT1G71380), DUF936 (AT2G31920)	TAP	Van Leene *et al.*, 2010

* Triple Complex

The Armadillo BTB *Arabidopsis* Protein 1 (ABAP1) is a plant-specific protein that binds directly to ORCa/b and CDT1a/b in *A. thaliana*. ABAP1 negatively regulates the assembly of the pre-RC, which reduces DNA replication licensing and cell division rates (Masuda *et al.*, 2008). ABAP1 could possibly prevent the assembly of a functional pre-RC complex by directly binding to members of pre-RC and/or by formation of a complex with TCP24, a transcription factor belonging to TCP class II that represses transcription of *CDT1a* and *CDT1b* (Masuda *et al.*, 2008). ABAP1 seems to participate in a negative feedback loop balancing mitotic DNA replication during leaf development (Figure 2).

Another pre-RC partner is Glabra2-Expression Modulator (GEM), that binds CDT1a and CDT1b and is a candidate to play an important role controlling cell specification in *A. thaliana* (Caro *et al.*, 2007). GEM promotes the differentiation and specification of root hair, probably by methylation of histone H3K9 to control expression of genes responsible for cell fate decisions in root (Caro *et al.*, 2007) (Figure 2).

### Protein stability and subcellular distribution

The entrance into the cell cycle must be finely coordinated as it responds to diverse types of stimuli, including different growth factors, such as hormones and nutrients (Inzé and De Veylder, 2006; Takatsuka and Umeda, 2014). The majority of these regulators are connected to the cell cycle through the activity of cyclin-dependent kinases (CDKs), the main actors regulating progression through the cell cycle (Van Leene *et al.*, 2010). CDKs work as a complex with cyclins for further phosphorylation of specific targets, in a general mechanism that involves: (1) cyclin synthesis and destruction, (2) CDK-cyclin assembly, (3) inhibitory and activating phosphorylation, and (4) the binding of inhibitory proteins (Inzé and De Veylder, 2006) (Figure 2).

Once the pre-RC is assembled in replication origins, a series of phosphorylation on specific targets regulates pre-RC activation followed by prevention of re-replication. Several members of the pre-RC are reported to be CDK regulation targets, such as CDC6, ORC1, ORC3 and MCMs; and the resulting phosphorylation of these proteins drives them to different fates (Errico *et al.*, 2010). In budding yeast, after assembly of pre-RC and licensing of DNA, ScORC (possibly ScORC2 and ScORC6) and CDC6 are phosphorylated by CDC7-DBF4 and the cyclin dependent kinase CDC28 (cdc2)-CLN1 (Jackson *et al.*, 1993). This is followed by the displacement of CDC6 and MCM10 binding for activation of MCM 2–7 and initiation of DNA replication. CDC7/DBF4 kinases are as well important in regulating animal pre-RC, but they are not described in plants so far (Scofield *et al.*, 2014).

To prevent re-replication, both in budding yeast and in animals, phosphorylated CDC6 becomes a target of the SCF proteasome machinery, and any remaining CDC6 is phosphorylated and inactivated by interaction with cyclins (Mimura *et al.*, 2004). In *A. thaliana,* phosphorylation sites in CDC6 (Ramos *et al.*, 2001) and MCMs (Dresselhaus *et al.*, 2006) were demonstrated, supporting a conserved regulation of the activation and prevention of re-replication steps in plants. To avoid re-replication, plants have as a key regulatory element the CDC6 dissociation from the pre-RC followed by its degradation, as well as the increase in CDC6 expression during S-phase and repression in G2 (Dambrauskas, 2003). During plant endoreplication, CDC6 is not degraded, and its overexpression induces rounds of endocycles (Bryant, 2011).

On the other hand, expression of most plant MCMs is greatly spread during the entire cycle (Springer *et al.*, 2000; Holding and Springer, 2002; Huang *et al.*, 2003; Cho *et al.*, 2008). It seems that plant strategies to prevent extra rounds of DNA replication based on MCM regulation seem to be much more similar to animals than to budding yeast. MCM5 and MCM7 are the MCMs that topologically load onto DNA in plants (Shultz *et al.*, 2009). MCM7 changes its dynamic of localization during the cell cycle, where it is highly associated with chromatin in phases G1, S and G2, and is dispersed in the cell only in the M phase (Shultz *et al.*, 2009). This indicates that plants do not regulate origin licensing by actively exporting the MCM complex from the nucleus during S phase, like in yeast. In *A. thaliana* and *Nicotiana benthamiana,* MCM5 and MCM7 are in the nucleus throughout most of the cell cycle and are displaced for only a brief period during mitosis (Shultz *et al.*, 2009). Plant ORC1 is weakly associated to chromatin (Shultz *et al.*, 2009), compared to mammals, where ORC1 is not part of the core origin recognition complex (Ohta *et al.*, 2003).

At the transition between M and G1 in many eukaryotes, MCMs are transported back to the nucleus to take part in another round of DNA replication licensing (Blow and Prokhorova, 1999). In animals, however, they remain inside the nucleus, and it is the inactivation of CDT1 that prevents MCM to load onto DNA again, as CDT1 is a key factor during MCM recruitment (Blow and Dutta 2005; Kerns *et al.*, 2007; reviewed by Rizzardi and Cook 2012). CDT1 is regulated mainly by ubiquitin-targeted proteolysis in animals (a process triggered by CDK/cyclin activity) and by interaction with Geminin, which sequesters CDT1, avoiding the complete pre-RC re-assembly (Suchyta *et al.*, 2015). Geminins have also been implicated in the regulation of cell proliferation and cell differentiation by interacting in a competitive way with components of the pre-RC (CDT1) or with transcription factors (Six and Hox) (del Bene *et al.*, 2004; Luo *et al.*, 2004). Unlike animals, plants do not seem to have a Geminin homolog, and it is not yet determined if and how CDT1 regulates MCM loading. The CDT1 partner GEM shares similarity with animal Geminins, as the competition of GEM for binding to CDT1 or to the transcription factor TTG1 might modulate the expression of the homeobox factor GLABRA (GL2) and control cell division (Caro *et al.*, 2007). GEM is an ABA-responsive protein that has a role in seed dormancy (Mauri *et al.*, 2016); nevertheless the function of GEM in regulating the availability of CDT1 and DNA replication licensing in plants remains to be demonstrated.

## Regulation of pre-RC licensing by mRNA expression profiles.

The spatial and temporal expression patterns of the pre-RC genes have been studied in different plant species using various experimental approaches, such as in *O. sativa* (Kimura *et al.*, 2000; Mori *et al.*, 2005; Cho *et al.*, 2008); *Z. mays* (Sabelli *et al.*, 1996, 1999; Bastida and Puigdomenech, 2002; Witmer, 2003; Li *et al.*, 2005; Dresselhaus *et al.*, 2006); *A. thaliana* (Witmer, 2003; Collinge *et al.*, 2004; Masuda *et al.*, 2004; Raynaud *et al.*, 2005; Shultz *et al.*, 2007) and *N. tabacum* (Dambrauskas, 2003). The responses of pre-RC gene expression to various signals indicate that transcriptional control might exert an important level of regulation of the complex function. In addition, the data showed different expression profiles among pre-RC components suggesting the existence of various forms of the complex, possibly playing different roles during development (Raynaud *et al.*, 2005; Dang *et al.*, 2011). Finally, the expression of pre-RC genes in tissues with low levels of cell proliferation suggests that they are likely to perform functions outside of the complex, not related to cell division (Dresselhaus *et al.*, 2006; Dang *et al.*, 2011). Various studies have also identified pre-RC interactions with proteins not related to the plant cell cycle (Table 2).

Analyses of mRNA levels of pre-RC members from *A. thaliana* during a cell cycle round revealed a cell cycle-regulated profile, as transcript levels of pre-RC members are reduced significantly during S phase, especially ORC3 and CDC6 (Castellano *et al.*, 2001; Ramos *et al.*, 2001; Masuda *et al.*, 2004). Later, in the transition from G2/M, mRNA levels of most members rise again, displaying a similar pattern (Castellano *et al.*, 2001; Masuda *et al.*, 2004). However, the cell cycle expression profile of pre-RC members varies among plant species. In *N. tabacum* cell cultures, *NtMCM3* expression is not confined to a cell cycle-specific phase, differently from *NtCDC6*, which is cell cycle regulated (Dambrauskas, 2003). In rice, MCM5 and -7 are found in high levels in the nucleus during both G1/S and G2/M phases (Shultz *et al.*, 2009). MCM6 expression is high, but it declines during S phase to undetectable levels in late S and G2 phases in maize (Dresselhaus *et al.*, 2006).

Possibly, the cell cycle activation of pre-RC genes transcription in G1 is mainly mediated by the RBR-E2F pathway, a major activator of the cell cycle machinery that is highly conserved in eukaryotes, including the plant kingdom, from macro- and microalgae to eudicots (Bisová and Zachleder, 2014; Desvoyes *et al.*, 2014; Huysman *et al.*, 2014). The phosphorylation of RBR by the CDK-cyclinD complex is a trigger point that releases the expression of cell cycle genes that are under the control of E2F-DP, such as pre-RC members (reviewed in Scofield *et al.*, 2014), and irreversibly commits the cell to DNA replication (Dimova and Dyson, 2005) (Figure 2). Promoters of all *A. thaliana* pre-RC genes, excluding *AtORC5*, have a putative E2F consensus binding motif (Castellano *et al.*, 2001, 2004; Diaz-Trivino *et al.*, 2005). It was shown that the *CDT1a* and *ORC2* genes are regulated before S phase by E2F in *A. thaliana* (Castellano *et al.*, 2004; Magyar *et al.*, 2012).

The spatial and temporal mRNA localization of different members of the pre-RC machinery can be very diverse, depending on the tissue observed, and their regulation might also involve epigenetic control besides transcription factors (reviewed in Raynaud *et al.*, 2014). For example, the function of *A. thaliana* DNA replication factor C was recently described in the maintenance of gene silencing through histone methylation (Liu *et al.*, 2010). Furthermore, at the G1/S transition, histone acetylation is required for the specification and activation of replication origins (Costas *et al.*, 2011). A new Agenet/Tudor domain protein named AIP1 (ABAP1 interacting protein 1) was described as interacting with histones, ABAP1, and the plant histone modification reader (LHP1) to regulate the expression of ABAP1 and LHP1 target genes in flower buds, such as *AtCdt1b* and *Flower Locus T (*FT*),* respectively (Brasil *et al.*, 2015). In addition, intrinsic or extrinsic signals may affect plant growth, interfering with cell cycle regulation, endoreduplication, stress responses and cell death (Diaz-Trivino *et al.*, 2005; reviewed in John and Qi, 2008; Magyar *et al.*, 2012; Sabelli *et al.*, 2013). All these processes may be part of the same machinery, directly coordinating the expression of pre-RC members.

Consistent with a role in DNA replication, mRNAs of *ORCs* from *A. thaliana*, rice and maize, as well as transcripts of *AtCDC6*, *AtMCMs* and *ZmMCMs* are abundant in proliferating tissues, such as root tips, lateral root development, seedlings, young leaves and flower buds (Sabelli *et al.*, 1996; Springer *et al.*, 2000; Castellano *et al.*, 2001; Bastida and Puigdomenech, 2002; Masuda *et al.*, 2004; Diaz-Trivino *et al.*, 2005; Schreiber *et al.*, 2006; Dang *et al.*, 2011; Chen *et al.*, 2013). In rice, *OsORC1* and *OsORC2* are strongly expressed in roots and inflorescence meristems, respectively, but there is a reduction of expression of *OsORCs* in tissues with low proliferative rates, leading to complete absence in mature leaves (Kimura *et al.*, 2000; Li *et al.*, 2005). Similarly, mRNA levels of maize *ZmORCs*, except for *ZmORC3*, are very low in mature leaves with few dividing cells, differing from the high expression levels observed in highly proliferative tissues (Witmer, 2003; Schreiber *et al.*, 2006; Sabelli *et al.*, 2013).

In *A. thaliana,* pre-RC members can also be found in organs with low cell division rates (Masuda *et al.*, 2004; Diaz-Trivino *et al.*, 2005). This expression profile outside proliferating cells could be correlated with DNA replication in endoreduplicating cells (reviewed in John and Qi, 2008). At*ORC1a,* and not *AtORC1b,* is highly expressed in tissues that undergo extra endocycles of DNA replication (Diaz-Trivino *et al.*, 2005). The two *AtCDC6* genes also present different expression profiles (Masuda *et al.*, 2004), and *AtCDC6a* expression in non-dividing tissues has been associated with endoreduplication events (Castellano *et al.*, 2001). Also, higher AtCDT1 levels are directly correlated with an increase in the number of endocycles (Castellano *et al.*, 2004).

Alternatively, pre-RC members could also be involved in regulating processes other than licensing DNA for replication. In other eukaryotes, pre-RC proteins have been implicated in different roles, such as transcriptional regulation and heterochromatin assembly in yeast and metazoans, sister chromatin cohesion, chromosome segregation and cytokinesis, neuronal development, and centriole and centrosome copy number (Hemerly *et al.*, 2009; reviewed in Sasaki and Gilbert, 2007). Functional data have already demonstrated that the role of pre-RC members in plants is not limited to licensing DNA for replication, but is also related to the regulation of gene expression, chromatin condensation and cell differentiation, directly influencing the growth and development of plants from embryogenesis until the senescence (discussed below – see Table 3).

**Table 3:** Phenotypes of pre-replication complex (pre-RC) mutants in plants available in the literature. Mutant plant with loss of gene function are in red squares; overexpression or ectopic expression are in green squares.

Genes	Vegetative Phase	Reproductive Phase	Endoreduplication	Others	Reference
ORC1		Single mutants are lethal in *A. thaliana*		*A*ctivation of expression of pre-RC genes through epigenetic regulation in *A. thaliana*	De La Paz Sanchez and Gutierrez, 2009
ORC2	Cell cycle arrest by abnormal cell divisions in *A. thaliana*	Chromatin instability leading to embryo abortion in *A. thaliana*			Collinge *et al.*, 2004
ORC3	Defects in lateral root development in *O. sativa*				Collinge *et al.*, 2004
ORC6		Abortion in G1/S phase of the generative cell during male gametogenesis in *A. thaliana*			Qin *et al.*, 2014
MCM2	Promotion of lateral root initiation close to the root tip in *A. thaliana*		Reduced growth and inhibition of endoreduplication in early embryo stage in *A. thaliana*		Ni *et al.*, 2009
MCM6	Demonstration of full helicase activity by itself in *P. sativum*			Promotion of salt tolerance, possibly by activating the expression of stress-related genes, in *P. sativum* and *N. tabacum*	Dang *et al.*, 2011; Quang *et al.*, 2010
MCM7		Defects in ovule formation in *A. thaliana*			Herridge *et al.*, 2014
MCM2-7 (single subunits)		Seed abortion in early and late stages in *A. thaliana*			Herridge *et al.*, 2014
CDT1a		Abortions by defects in egg cell development in *A. thaliana*	Increase in nuclear ploidy during leaf development in *A. thaliana*		Domenichini *et al.*, 2012; Castellano *et al.*, 2004
CDT1a/CDT1b double mutant	Delayed growth and yellow leaves and maintenance of the repressor histone H3K9 methylation status of root patterning genes in *A. thaliana*	Seed abortions due to a role in female gametophyte in *A. thaliana*	DNA stress leading to genome instability probably due to incomplete genome replication during S-phase in *A. thaliana*		Domenichini *et al.*, 2012; Caro *et al.*, 2007
CDC6				Extra endocycles in *A. thaliana*	Castellano *et al.*, 2001

Plants seem to sense many environmental conditions and adjust cell cycle progression, at least in part, by regulating the expression of pre-RC components. In this context, plant hormones have great importance by integrating signaling between organs and tissues within the plant and among other organisms, and in response to the environment (Takatsuka and Umeda, 2014). In higher plants, the TOR kinase protein integrates the auxin pathway with nutrient signals, resulting in both growth and cell division responses to the environment (Bögre *et al.*, 2013; Henriques *et al.*, 2014). In cell cultures, auxin has been implicated in participating in the stabilization of *E2Fb*, compromising cells to the G1 to S phase transition (Magyar *et al.*, 2005; Berckmans *et al.*, 2011). Cell cycle progression is also regulated by sucrose, which stimulates progression into S phase and cell division by controlling cyclins and pre-RC gene expression (Masuda *et al.*, 2004; Planchais *et al.*, 2004, Menges *et al.*, 2006). Recently it was suggested that methyl jasmonate contributes to inhibit the initiation of DNA replication by repression of CDC6 and CDT1 levels (Noir *et al.*, 2013).

## Epigenetic mechanisms in pre-RC function.

Although not yet well understood, animal DNA replication timing and its regulation through epigenetic mechanisms, ranging from DNA methylation to histone modifications and higher order chromatin structure, have been studied more deeply in the last years (reviewed by Casas-Delucchi and Cardoso, 2011). In plants, 130 epigenetic regulators have been described so far, revealing enormous diversity and contributing to the understanding of plant plasticity (Pikaard and Mittelsten Scheid, 2014). However, very little is known about epigenetic mechanisms regulating the cell cycle.

As discussed above, some epigenetic marks are enriched in DNA replication origins of *A. thaliana* (Costas *et al.*, 2011), suggesting that chromatin modification proteins could be involved in specifying replication origins and/or regulating DNA replication timing in plants.

ORC1 has an interesting role in the activation of pre-RC through epigenetic regulation of gene expression. The role of BAH domains of ORC1 in transcriptional silencing has been well studied in yeast (Rusche *et al.*, 2003), and their involvement in DNA damage was reported in metazoans (Kuo *et al.*, 2012). In *A. thaliana*, the PHD domain, present in the C-terminus of both *ORC1* genes, is responsible for the recognition and binding to histone H3K4me3 residues in promoters of target genes such as *MCM3*, *CDT1a*, and *ORC3* (Sanchez and Gutierrez, 2009). Moreover, the binding of ORC1 to its targets is related to the increase in H4 acetylation and trimethylation of H4K20 (Sanchez and Gutierrez, 2009). Recently, the crystal structure of the AtORC1b BAH-PHD cassette and its preference for the unmodified state of key amino acids when in complex with a H3 peptide was reported, revealing a unique “sandwiching” mode of ORC1 recognition of its targets (Li *et al.*, 2016).

The Agenet/Tudor-domain protein, AIP1, which interacts with the pre-RC regulator ABAP1 and with unmodified histones, regulates the expression of ABAP1 target genes such as *AtCdt1b* and seems to connect DNA replication and transcription to chromatin remodeling controls during flower development (Brasil *et al.*, 2015).

Therefore, pre-RC members, pre-RC regulators and epigenetic players were described as partners in regulating both transcription and replication origin specification and/or activation in plants. Transcription and replication are two cellular processes that require chromatin accessibility, however it has not yet been elucidated if and how they are integrated in plant cells. As discussed above, a high percentage of origins in *A. thaliana* co-localizes with the 5’ end of gene units and are more concentrated in highly expressed genes (Costas *et al.*, 2011). Hence, one question that remains to be answered is whether pre-RC proteins and their regulators, together with epigenetic mechanisms, could be, to certain extent, shared between replication licensing and transcription machineries to co-regulate these vital cellular processes.

## What do mutants tell us about pre-RC function?

Functional analyses of pre-RC components were investigated in plants with modified levels of gene expression. The observed phenotypes for each mutation are summarized in Table 3. These studies showed that mutants in plant pre-RC genes could interfere in developmental processes either by directly affecting replication or by indirect effects on endoreduplication and heterochromatin formation (Table 3).

Complete knockout plants for *ORC* genes cannot survive, indicating that these genes are vital for the plant to complete its life cycle (Collinge *et al.*, 2004; Sanchez and Gutierrez, 2009; Chen *et al.*, 2013). Constitutive expression of a specific mutation in the PHD domain of ORC1b in *A. thaliana* leads to the loss of the *ORC1b* overexpression phenotype characterized by increased cell proliferation (Sanchez and Gutierrez, 2009), revealing that PHD is essential for ORC function in DNA replication. ORC1 is not only required to form the pre-RC and to license DNA replication, but it also has a role in pre-RC activation through epigenetic regulation of gene expression (Takayama *et al.*, 2000; Saitoh *et al.*, 2002; Rusche *et al.*, 2003).

In *A. thaliana,* the *ORC2* mutants show a zygotic-lethal phenotype, with abortion of embryos in the early globular state and enlarged endosperm nuclei (Collinge *et al.*, 2004). It has been proposed that low *ORC2* levels in the mutants prevent pre-RC assembly and the lack of the check point before S-phase in embryo cells; this (a) leads to many endoreduplication cycles and the enlargement of the endosperm nuclei; as well as (b) triggers cell cycle arrest, leading to abnormal divisions and chromatin instability that results in abortion of the embryo (Collinge *et al.*, 2004).

In a developmental context, OsORC3 knockdown rice plants lack lateral roots and have a dwarf phenotype (Chen *et al.*, 2013). A very fast differentiation of lateral root cells requires rapid cycles of cell division for tissue maintenance and development. However, in cells with lower OsORC3 availability, the G1/S phase is compromised, leading to lower proliferation levels and tissue defects (Chen *et al.*, 2013).

*AtORC6* is required for the mitosis that gives origin to the two sperm cells of the *A. thaliana* male gametophyte, and its absence causes abortion of the generative cell, with aberrant DNA content (Qin *et al.*, 2014). proBRP4:ORC6 RNAi mutants with reduced expression of ORC6 display pollen developmental defects, and aborted generative cells have a lower DNA content, suggesting impairment at the G1/S phase (Qin *et al.*, 2014). ORC6 acts downstream of BRP4, a TFIIB-related protein involved in regulation of the mitotic cell cycle progression during male gametogenesis (Qin *et al.*, 2014).

AtCDT1a seems to have a specific function in female gametogenesis during embryo sac development in *A. thaliana*, while a majority of pollen grains harboring the *cdt1a* mutation develop normally, possibly due to a partial redundancy with AtCDT1b in the male gametophyte (Domenichini *et al.*, 2012). Nowack *et al.* (2006) first suggested that the storage of *CDK* mRNAs in female tissues would avoid proteolysis and maintain sufficient supplies for cell cycle activities, proposing that pre-RC proteins may accumulate in maternal tissues. Although not yet described for other members of the pre-RC, it is possible that *CDT1a* accumulates in higher levels than *CDT1b* at gametophyte development (Domenichini *et al.*, 2012) due to a CDT1b plastid-targeting sequence (Raynaud *et al.*, 2005). CDT1 and CDC6 are expressed in stomatal precursor cells, and overexpression of these genes increases the number of stomata in *A. thaliana* leaves (Castellano *et al.*, 2004). In addition, simultaneous silencing of both *Cdt1* genes shows a delay in cell cycle progression and an increase in endoreduplication (Raynaud *et al.*, 2005). Plants with double mutation in *CDT1a* and *CDT1b* show a variety of phenotypes, including: drastically reduced stature and deformed leaves; evidences of the DNA stress, probably due to incomplete genome replication during S-phase; aberrant DNA content and induction to cell death; better tolerance to DNA-damaging agents; and increased expression of genes involved in DNA repair (Domenichini *et al.*, 2012). Moreover, CDT1 interacts with the GEM protein and may participate in the maintenance of the repressor mark histone H3K9 methylation status on root patterning genes (Caro *et al.*, 2007).

Disruption of *A. thaliana* *MCM2* is lethal, causing plants to show problems at early embryo stage, while its overexpression in *A. thaliana* increases cell division in root meristems and decreases endoreduplication (Ni *et al.*, 2009). *mcm7* mutants show leaky lethality in the megagametophyte (Springer *et al.*, 1995), and its effects in embryo and endosperm development may be due the gene’s primary function in the ovule (Springer *et al.*, 2000; Holding and Springer, 2002).

*AtMCM7* was the first MCM protein identified in plants, reported with the name of *PROLIFERA* (*PRL*) and with functions in reproduction and embryo development (Springer *et al.*, 1995). Later, Herridge *et al.* (2014) studied all *MCMs* mutants separately during *A. thaliana* seed development. It was observed that, in general, reduced MCM in the endosperm promotes the expression of genes involved in DNA damage response in the ATM-dependent pathway. As expected for pre-RC genes, homozygous mutants for all *MCMs* are lethal, but different proportions of seed abortion in early and late stages are observed when heterozygous mutants are analyzed: *mcm2* and *mcm5* have the highest rates of late abortion and *mcm3* and *mcm4* the lowest. The endosperm defects in mutants are generally characterized by fewer and larger nuclei compared to wild-type seeds, the absence of cellularization in endosperm, and abnormal embryo cytokinesis. Among all mutants, *mcm7* is the more distinct one, and its defect at an early embryo stage confirms previous data that MCM7 is required during ovule development (Herridge *et al.*, 2014). The first evidence of a single subunit of *PsMCM6* with a full helicase activity *in vitro* was demonstrated by Tran *et al.* (2010). Helicase activity, as well as an ATPase activity, have been described for AtMCM3 with greater unwinding efficiency with 5-forked DNA, and the indispensable presence of ATP and magnesium ion (Rizvi *et al.*, 2016). All together, these data points out the difference between plant and animal MCM mechanisms of action, once multi-heterodimers of MCM were reported in animals only.

Interestingly, a role of pre-RC on environmental responses was shown by the overexpression of *PsMCM6* in *P. sativum,* which increased salinity tolerance and cold stress in an ABA-independent pathway (Dang *et al.*, 2011). While the exact mechanism of stress tolerance is not known, the *PsMCM6* characterization as Zn-HD family member supports its role in stress tolerance (Tran *et al.*, 2010).

Remarkably, deregulation of expression (silencing or overexpression) of the different pre-RC members show different effects on plant developmental processes, suggesting that they might have distinct roles in the pre-RC and/or might reflect their particular roles outside the complex. All together, the functional analysis data confirm that pre-RC genes are not only main actors in licensing DNA for replication, but that they additionally have a role in chromatin stability and as regulators of gene expression, either by interaction with transcription factors or by epigenetic pathways. These combined roles might act by integrating cell cycle progression in plants with developmental and environmental signaling.

## Conclusions and Perspectives

Plants are continuously sensing the environment and modulating their development by adjusting the growth, architecture and timing of development of new organs. This means that every plant meristem might be sensing exogenous signals and integrating these with genetic controls, which leads to changes in gene expression that will finally balance cell proliferation and differentiation rates, to generate the correct plant form. An important control of the G1 to S transition of the cell cycle is the pre-RC that is assembled at replication origins (Figure 1).

The identification of pre-RC members in all plant species shows that this complex is conserved among eukaryotes. More importantly, the differences seen between kingdoms might reflect their divergence in strategies for cell cycle regulation, as these must be integrated and adapted to the niche, ecosystem, and the organisms peculiarities. On the other hand, the high conservation of pre-RC components through evolution allows some of the discoveries in the plant systems to be translated to other multicellular organisms. The main discoveries on the regulation and biochemical mechanisms of DNA replication in multicellular eukaryotes have been made at the cellular level using animal systems. However, in multicellular organisms, cells belonging to different organs and tissues might respond to distinct developmental controls, that ultimately will modulate cell divisions in particular ways. Therefore, the facilities available to study plants as whole organisms make them a suitable model for developmental studies.

There are still very important aspects of the pre-RC regulation and activity that need to be elucidated in plants. The understanding of the mechanism of origin recognition and selection, and how it is integrated with transcription controls, could enlighten ideas on specific strategies of cell cycle regulation as well as how cell cycle controls respond to environmental changes, and finally, how plant plasticity is integrated with these processes. Although pre-RC regulators were identified and mutant studies gave insights on pre-RC functions, it is now necessary to assemble all the pieces of the puzzle to understand how cell cycle regulation is integrated with developmental and environmental signals, in order to generate adaptive responses, culminating with plant bodies adjusted to their surroundings.
